# Supplementary material for: The interactome of intact mitochondria by cross-linking mass spectrometry provides evidence for coexisting respiratory supercomplexes
Source: Mol Cell Proteomics. 2017 Dec 8;17(2):216–32. doi: 10.1074/mcp.RA117.000470 (PMC5795388; doi:10.1074/mcp.RA117.000470)
Supplement: Supplemental Data [file supp_RA117.000470_133922_0_supp_23978_fzffwf.zip › spectra_annotation/mito_DR_spectra_annotation/487-1-1-1-16-1.pdf]

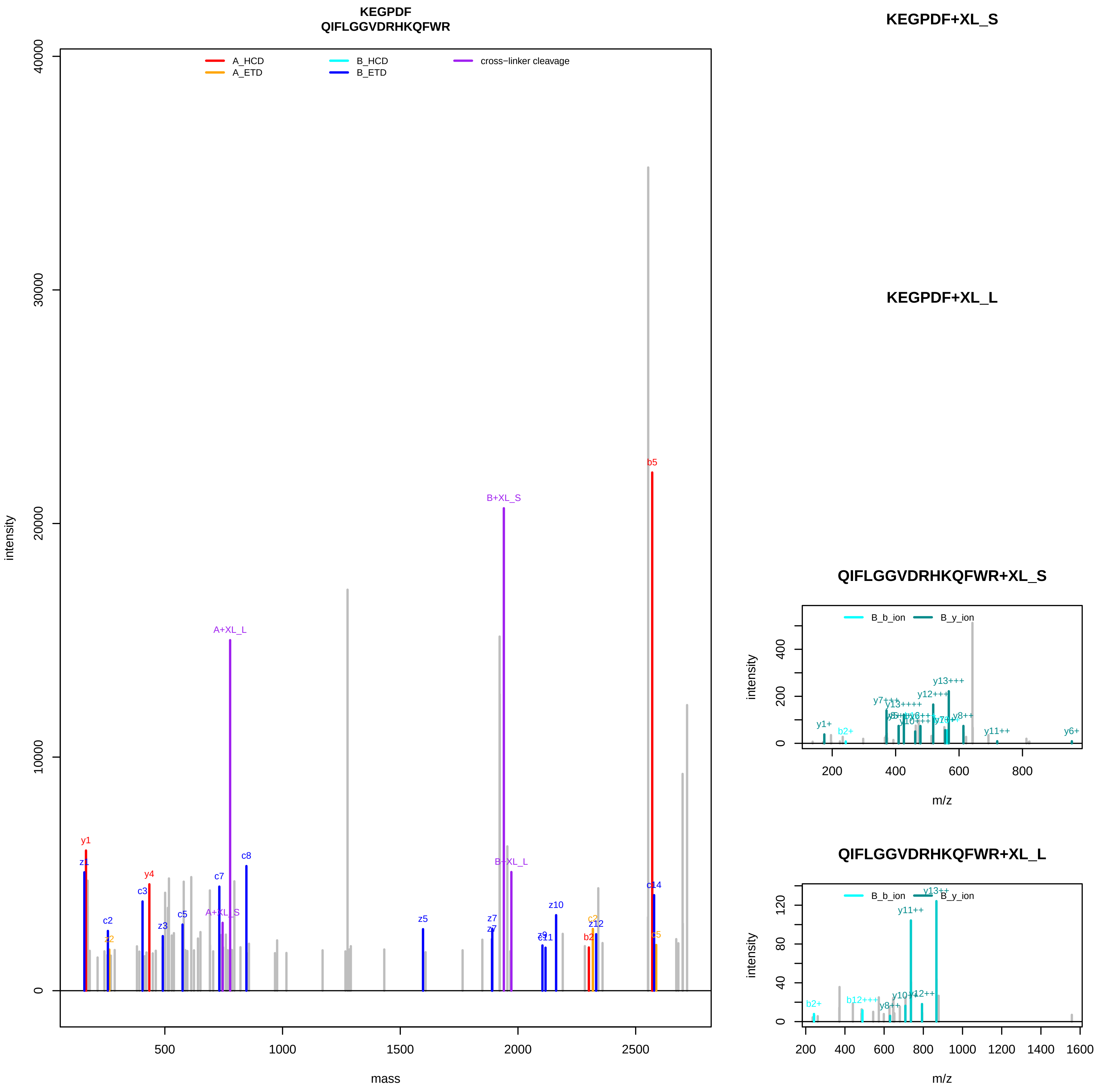
KEGPDF
QIFLGGVDRHKQFWR
A_HCD
A_ETD
B_HCD
B_ETD
cross−linker cleavage
intensity
mass
0
10000
20000
30000
40000
500
1000
1500
2000
2500
y1
z1
c2
z2
c3
y4
z3
c5
c7
A+XL_L
A+XL_S
c8
z5
z7
B+XL_S
B+XL_L
z9
c11
z10
b2
c2
z12
b5
c14
c5
KEGPDF+XL_S
KEGPDF+XL_L
QIFLGGVDRHKQFWR+XL_S
B_b_ion
B_y_ion
intensity
m/z
0
200
400
600
800
y1+
b2+
y7+++
y13++++
y12+++
y13+++
y8++
y11++
y6+
QIFLGGVDRHKQFWR+XL_L
B_b_ion
B_y_ion
intensity
m/z
0
40
80
120
200
400
600
800
1000
1200
1400
1600
b2+
b12+++
y8++
y10++
y12++
y11++
y13++
